# 8q22.1 Microduplication Syndrome: Why the Brain Should Be Spared? A Literature Review and a Case Report

**DOI:** 10.1155/2018/3871425

**Published:** 2018-07-12

**Authors:** Antonella Gagliano, Erica Pironti, Francesca Cucinotta, Cecilia Galati, Roberta Maggio, Maria Ausilia Alquino, Gabriella Di Rosa

**Affiliations:** Department of the Adult and Developmental Age Human Pathology, Unit of Child Neurology and Psychiatry, University Hospital of Messina, Messina, Italy

## Abstract

Microduplication of chromosome 8q22.1 is mainly associated to Leri's pleonosteosis syndrome phenotype, an extremely rare autosomal dominant disease encompassing the GDF6 and SDC2 genes. To date, most of the authors focus their attention only on skeletal symptoms of the disease, and they do not systematically research or describe the co-occurrence of psychiatric illnesses or mental disorders with these muscular-skeletal diseases. In this report, we provide a description of an 8-year-old girl, with a positive family history for both skeletal malformations and bipolar disorders (BD). We suggest a possible association between Leri's pleonosteosis features and psychiatric symptoms. Furthermore, our report could be added to the large amount of reports that describe the correlation between genetic regions and disease risk for both psychiatric and rheumatological disorders.

## 1. Introduction

Microduplication of chromosome 8q22.1 is an extremely rare genetic disorder with prevalent muscular-skeletal phenotype due to defect of the GDF6 and SDC2 genes. The muscular-skeletal features merely configure Leri's pleonosteosis (LP) phenotype, a very rare disorder first described in 1921 [1]. Few multigenerational families have been reported so far, and the prevalence is estimated as <1/10,00,000 [2].

Leri's pleonosteosis was originally described as a rare congenital rheumatic disease frequently overlapping with other muscoloskeletal diseases [1]. The overexpression of these genes dysregulates proteins of the extracellular matrix synthesis and transforming growth factor- (TGF-) *β* pathway [3]. Currently, it is assumed that the heterozygous microduplication of chromosome 8q22.1, encompassing GDF6 and SDC2 genes, has a causative role in the rheumatic signs of the disease [3]. The growth/differentiation factor-6 (GDF6) is a member of the transforming growth factor-beta (TGF-*β*1) superfamily, required for normal formation of bones and joints in the limbs, skull, and axial skeleton [4]. Syndecan-2 (SDC2) gene is a member of the syndecan proteoglycan family who produces a transmembrane heparan sulfate proteoglycan. The syndecan-2 protein acts as an integral membrane protein playing a role in cell proliferation, cell migration, and cell-matrix interactions [5]. More recently, Western blot analysis also revealed markedly decreased inhibitory SMAD6 (mothers against decapentaplegic homolog 6) levels in patients with LP [3]. SMAD6 is an antagonist of signaling by TGF-beta-type 1 receptor superfamily members and acts as a mediator of TGF-beta and BMP anti-inflammatory activity [3].

The LP clinical phenotype is characterized by dismorphic facial features, abnormalities of hands and feet, skeletal malformations, short stature, and limitation of joint movements. Skeletal malformations can include brachydactyly, “spade-shaped” appearance, genu recurvatum, and abnormal enlargement of the cartilaginous structures that surround the upper portion of the spinal cord [6]. A review of the literature with a detailed report of four cases was provided by Watson-Jones in 1949 [7]. The author focused his attention only on skeletal symptoms of the disease. Around ten years later, Yeoman described the case of a 16-year-old girl having the muscular-skeletal features of the disease with no cognitive impairment (he described her as an “intelligent girl”) [8]. A few years later (1959), Rukavina et al. reported a four-generation family with LP [9], and Booth observed a father and his son with the same condition [10]. In the 1980s, Hilton and Wentzel reported seven patients from the same family [11], and Friedman described two single cases [6]. Approximately 30 cases have been reported in the literature at the current state.

Most of the authors reported LP cases focusing their attention only on the somatic features (e.g., facial dimorphic features and skeletal and joins malformations). To the best of our knowledge, the only detailed description of psychiatric symptoms in LP was provided by Macayran et al. [12]. They presented a 7-year-old boy affected by LP and bipolar disorder. The child showed a speech-sound disorder and a highly pressured pattern of speech. He also displayed difficulty in sustaining attention, high levels of hyperactivity, low frustration tolerance, indiscriminate friendly and socially inappropriate behaviour toward adults. The cognitive assessment was performed by the Kaufman Assessment Battery for Children (K-ABC) [13] that showed a “mental process composite” score of 86 (−1 DS) at the age of 5 and 6. Nevertheless, one and a half years later, the mental process composite score decreased to 74 points (−2 DS). Thus the authors assumed that the patient had had a cognitive functioning regression. Additionally, he showed decreased need for sleep, flight of ideas and increased sexualized behaviour, in opposition to previous depressed mood episode. Cytogenetic analysis revealed a 46, *XY*, ins (15;8) karyotype with a duplication of 8q, confirmed by chromosome 8-specific subtelomere FISH analyses. Recently, a more complex case regarding a 14-year-old boy presenting both 142 Kb duplication in 8q22.1 and 252 Kb duplication in 22q11.2 was reported. The patient showed mild cognitive impairment (IQ 70) and attention deficit disorder. However, the presence of a double duplication in two different genomic regions makes it difficult to correlate the psychiatric features to the 8q22.1 duplication [14].

In the past few years several studies documented the systematic co-occurrence of psychiatric illnesses mental disorders and systemic diseases [15]. In particular, many genome-wide association studies (GWAS) have been conducted to identify genetic risk variants underlying psychiatric disorders [16]. Meanwhile the epidemiological observation of a frequent co-occurrence of autoimmune disorders such as rheumatoid arthritis (RA) and depression has focused the attention on genetic background underlying these comorbidities [17–19]. Furthermore, strong evidence for the existence of a genetic relationship and a pervasive pleiotropy between psychiatric disorders and immune disorders have been collected [20].

The present work describes an 8-year-old girl that showed a Leri's pleonosteosis phenotype associated with neurodevelopmental disorders and psychiatric symptoms.

## 2. Case Description

Written informed consent for publication was obtained by the patient's parents.

**Figure 1:**
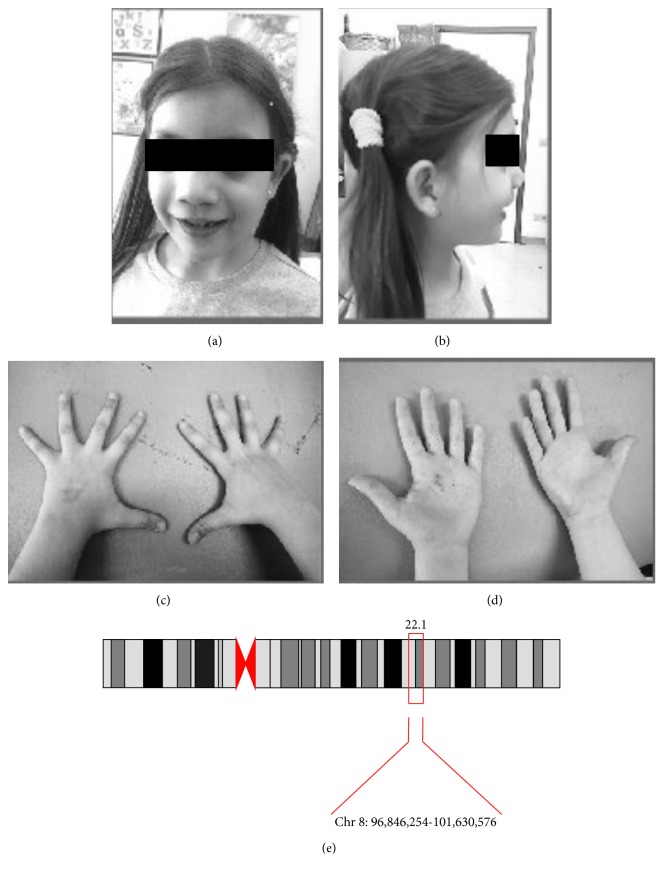
(a, b) Facial picture of the 8-year-old girl showing facial dimorphism: flat face, blepharophimosis, hypertelorism, broad nasal bridge, and high palate. (c, d) Hands photograph showing bones and joints defects: single transverse palmar crease and brachydactyly. (e) The 8p22.1 microduplication and its coordinates in our patient.

Our patient is an 8-year-old girl, with a positive family history for both skeletal malformations and bipolar disorders (BD). Her pre-perinatal history was uneventful. She was referred to our Unit because of learning difficulties and behavioural problems. The neurological examination did not show focal neurological deficits. Dysmorphic features were evident at the first observation. She showed several facial dimorphisms such as flat face, blepharophimosis, hypertelorism, broad nasal bridge, and high palate. Bones and joints defects were also evident: pectum excavatum, single transverse palmar crease, brachydactyly, flat foot, and stature below 25th percentile (Figure 1). Because of these features, she previously underwent genetic consultation and performed array-CGH analysis revealing a chromosomic 8q22.1-q22.3 duplication (hg19/96.846.254-101.630.576x3, 101.726.279x2) encompassing the GDF6 and SDC2 genes, inherited from her father. Thus, our 8-year-old girl presented with clinical and genetic features of Leri's pleonosteosis, within a larger microduplication involving different genes not strictly related to our patient phenotype. In particular, the hypothesis of autosomal recessive optic atrophy (OPA6) was excluded by a general ophthalmologic examination and a fundus examination, since the contiguous region 8q21.13-q22.1 is responsible for recessive optic atrophy [21].

She also met the DSM-5 criteria for attention-deficit/hyperactivity disorder (ADHD), specific learning disorder, speech sound disorder, and developmental coordination disorder. In particular, she showed a highly pressured pattern of speech, difficulty in sustaining attention, high levels of activity, and low frustration tolerance. Furthermore, she presented a pattern of bipolar-like phenomena that did not meet the criteria for bipolar I, bipolar II, or cyclothymic disorder. Nevertheless, according to DSM-5 category, she met the criteria for the diagnosis of “other specified bipolar and related disorder” owing to the occurrence of hypomania episode without prior major depressive episode or a manic episode.

The clinical features are shared with both her father and her grandfather that present an overlapping duplication in the 8q22.1-q22.3 region. They show facial dimorphism (flat face, blepharophimosis, hypertelorism, and broad nasal bridge) and brachydactyly and are affected, respectively, by cyclothymic disorder and bipolar II disorder. Also her grandfather's brother received a diagnosis of bipolar II disorder. Unfortunately, he has never performed an array-CGH analysis, but he shows skeletal malformations consistent with Leri's disorder. Along paternal line of our patient, more members are affected by mood disorders associated with skeletal deformations. Unfortunately, none of them agreed to perform the array-CGH analysis, thus the information is incomplete to build a family tree chart. Nevertheless, the chromosome 8q22.1 microduplications were documented in our patient and his father and grandfather.

Our 8-year-old girl's developmental milestones had been mildly delayed. In particular, she presented a delayed achievement of the expressive language. Thus she started a speech-language therapy when she was 4-year-old and continued it for two years. During infancy, she also presented a divergent strabismus surgically treated at the age of 3 years. She also had genu recurvatum and hip developmental dysplasia (type-II Graf) within the first year of life. Our first neurological examination failed to detect major focal signs, but gross and fine coordination impairments with orofacial dyspraxia and speech phonological deficits were observed.

The behavioural observation revealed high levels of impulsivity and a persistently elated mood with increased activity and energy for most of the day. She often displayed restlessness, hyperactivity, and difficulty remaining focused. She also was more talkative than usual and prone to engage conversations with strangers in public, with an indiscriminately friendly approach and a high level of enthusiasm. On the whole, her social behaviour towards adults was often inappropriate. Furthermore, the conversation content appeared inappropriate to the contest with flight of ideas and abrupt shifts from one topic to another. She also showed amusing irrelevancies and theatrical mannerisms and an inflated self-esteem with uncritical self-confidence. Moreover, rapid shifts in mood over brief periods of time might occur. During these episodes, her mood became irritable and she presented decreased need for sleep, diminished ability to concentrate, inconclusiveness, and angry bursts when her wishes were denied.

The neuropsychological assessment included the cognitive profile (WISC IV) [22], visual perception and motor coordination (VMI) [23], executive functions (TOL) [24], and verbal and spatial memory (Corsi test) [25]. The IQ score was in the low average (total IQ score 88), with the lowest score in nonverbal and fluid reasoning (perceptual reasoning index = 80). A moderate impairment was revealed in visuospatial short-term working memory (Corsi backward span = 2; −1.89 SD) and in backward verbal span (digit backward span = 2; −1.29 SD). Planning ability was largely below the average (rule violations = *T* > 100, >−2 SD; number of additional moves = *T* > 100, >−2 SD, TOL), suggesting a poor mental planning and problem-solving skills. She also showed deficits in visual perception and motor coordination skills at the visual-motor integration test (VMI Beery, 1997; standard score = 78; 7th percentile). The reading and writing tests scored below the average (oral reading speed of a text: 0.88 syllables/seconds/−2 DS; number of errors = 14/5° percentile; writing errors: −2 DS). She lacked knowledge of spelling rules that made her enable to read and write complex words. In general, she showed difficulties in managing the aspect of phonological processing which underpins the acquisition of literacy. Working out meaning from a whole sentence was another of her weaknesses due to the working memory deficit. Instead, when dealing with words in isolation, she had less difficulty in comprehension. She also showed very low concentration and attention abilities. During the assessment, she had to be constantly refocused and prompted to continue. All in all, our patient's neurodevelopment and psychiatric symptoms caused a marked impairment, especially in social relationship and in academic performance.

## 3. Discussion

This case report describes an 8-year-old girl with microduplication of chromosome 8q22.1 characterized by Leri's pleonosteosis features, neurodevelopmental, and psychiatric disorders. This peculiar genotype-phenotype profile is shared with some other members of her family that display both musculoskeletal and psychiatric symptoms, associated with the microduplication of chromosome 8q22.1. This feature labels the co-occurrence between psychiatric symptoms and rheumatic condition sustained by a genetic disorder. It is also noteworthy that the psychiatric profile of all members of our family largely coincides with that of Macayran et al.'s patients [12], who had a larger duplication than patients with definite LP with muscular-skeletal manifestations only. Moreover, the 14-year-old boy reported by Tarsitano showed a more complex phenotype, but he presented both 142 Kb duplication in 8q22.1 and 252 Kb duplication in 22q11.2. According to all these observations, even if our patient presents a larger microduplication of chromosome 8q22.1 encompassing additional genes, none of them strictly explains her neuropsychiatric symptoms. Indeed, although prominent muscular-skeletal features resembling LP syndrome emerged in our patient, the occurrence of a complex neuropsychiatric phenotype as well as a larger duplication than the sole occurring in definite LP patients should suggest a different syndromic entity.

The two key questions of the present report are the following:

1. Given that overexpression of GDF6 and SDC2 genes dysregulates proteins of the extracellular matrix synthesis and transforming growth factor (TGF)-*β* pathway, why should the brain be spared from damage?
2. Might the psychiatric comorbidity be underestimated in patients with LP features?

In recent years, an intensive research effort has focused on understanding the function of the TGF-*β* superfamily in midbrain dopaminergic neuron development and their role in the molecular architecture that regulates the development of this brain region [26]. Older studies have suggested that TGF-*β* 2 and TGF-*β* 3 are physiological survival factors for developing midbrain dopaminergic neurons [27]. Furthermore, the TGF-beta isoforms -beta2 and -beta3, members of the TGF-beta superfamily, are expressed in the central nervous system and have an important role in embryonic patterning, cell migration, and neuronal transmitter determination [28]. The formation of neuronal connections in the developing brain is regulated by interactions of the cell surface with extracellular matrix molecules, soluble growth factors, and cell surface adhesion molecules. There is evidence for a role of syndecan-3 in cell-matrix adhesion in the developing central nervous system [29]. It is assumed that syndecan-3 functions as an HB-GAM receptor in the central nervous system [30]. HB-GAM (heparin-binding growth-associated molecule), also called pleiotrophin, is an 18 kDa secreted protein that is expressed in the central nervous system with a developmental time course that is essentially identical with that of syndecan-3 [31]. Purified HB-GAM promotes the attachment of a variety of cells and is a potent inducer of neurite outgrowth from embryonic or early postnatal cortical neurons [32]. Syndecan-3 is also present on the surface of cortical neurons spreading on surfaces coated with HB-GAM [32]. For all of these reasons appears at least counterintuitive to suppose that the brain is not involved in LP disease.

Such a complex model of 8q22.1 microduplication syndrome could be better explained considering the developmental trajectories of mental-physical comorbidity and the temporal association of mental disorders and physical diseases. In our patient, the very early onset of psychiatric symptoms largely contributes to explain the severity of the LP disease. Many diverse underling factors, both genetic and environmental, could have contributed to the onset of psychiatric symptoms that represent in our patients the prominent features of the LP phenotype. Notably, many linkage studies have implicated chromosome 8q24 as a promising positional candidate region in BP [33], but no evidence has been reported on the association between BP and 8q22.1 region. Only a life course perspective, not focused on selected mental or physical problems, could consent to provide a more accurate description of the whole clinical features. Therefore, we argue that the psychiatric comorbidity of LP disease could be underestimated.

The report provides a description of a possible wider phenotype of Leri's pleonosteosis disease, encompassing not only the physical signs but also psychiatric symptoms, and supports the conception of a more integrated mental-physical health care approach. Further research is required to provide evidence of the association between rheumatic and psychiatric symptoms in LP. Nevertheless, the current report suggests that the psychiatric and neuropsychological LP patients' characteristics should be taken into consideration when making treatment decisions. Focusing the attention only on the rheumatic condition and musculoskeletal signs may lead to underrate the impact of psychiatric symptoms in the clinical management of these patients.

Furthermore, our report could be added to the large amount of previous reports that describe the correlation between genetic regions and disease risk for psychiatric and rheumatology disorders. The future research, particularly high-quality omics data, could provide an extraordinary opportunity to revisit the nature of the genetic connections between psychiatric and rheumatology disorders. More in general, the current understanding of the etiology of mental-physical comorbidity needs to be improved by theoretical models attempting to explain the trajectories of mental-physical comorbidity.
